# Common Fiscal Capacity Is Needed to Strengthen Risk Sharing

**DOI:** 10.1007/s10272-020-0905-1

**Published:** 2020-07-28

**Authors:** Antonia Díaz

**Affiliations:** Department of Economics, Universidad Carlos III de Madrid, Calle Madrid, 126, 28903 Getafe-Madrid, Spain

## Abstract

This asymmetric effect of the shock on economic activities becomes an asymmetric impact on countries depending on their sectoral composition.

The COVID-19 pandemic has shown that the fabric of the European Union project, in its current design, cannot facilitate the needed risk sharing to deepen the Single Market. To boost cross-border banking and capital market integration, we need a common safe asset and therefore common fiscal capacity.

* This article draws some ideas from Díaz (2020) and Díaz and Puch (2020).

## The heterogeneous effect of COVID-19 across sectors and regions in the EU

Until we have a successful vaccine or an antiviral drug, the COVID-19 shock remains a dramatic example of a negative externality: people may become infected when gathering for social or productive purposes. The very nature of the shock implies that people, depending on their economic activity, have different probabilities of getting infected. In particular, activities that require team production (i.e. an assembly line) or those services that require the joint time of the provider and consumer (a restaurant) are severely disrupted by the coronavirus pandemic and the slow-down measures required to prevent infections. Other activities, however, especially in information and communication sectors, are less affected by the pandemic as workers can move more easily to teleworking. This asymmetric effect of the shock on economic activities becomes an asymmetric impact on countries depending on their sectoral composition. For instance, this shock hits regions specialised in services such as tourism particularly hard.

Figure 1 shows a standard measure of employment specialisation across regions of the European Union. It reveals that regions with greater specialisation in retail trade are more affected by the pandemic. The impact of the shock is not only larger in regions specialised in contact-intensive services but, most likely, will have a more persistent effect. There are two reasons for this: first, substitution of labour for capital (robotisation) is much more difficult in non-routine jobs. Second, keeping safe physical distance implies that the very size of plants, shops, restaurants, etc. is a binding capacity constraint during the unlock phase. That is, restarting contact-intensive services in a way that complies with safety conditions requires a significant amount of investment in the sectors that have been hardest hit by COVID-19.

Moreover, the shift in consumer expenditures from face-to-face services to online services (or goods) implies that there are sectors that are actually benefitting from this crisis. In particular, the digital sector, dominated by international giants like Netflix, Google and Apple is making substantial profits in, almost literally, captive markets.

## Risk sharing is needed to deepen economic integration

COVID-19 is an example of a shock that hits households, firms and sectors asymmetrically, within and across countries. This is particularly important in the European Union since all measures taken to boost the Single Market lead to exploiting comparative advantages across regions and, thus, sectoral specialisation; especially in the eurozone, as Mongelli et al. (2016) show. This specialisation raises the exposure of any regional economy to some risks and, therefore, increases the variance of its GDP. It could be argued that the COVID-19 shock is an extremely unlikely event, but there are many studies that point out that differences in sectoral composition are in fact an important factor in explaining the size of the business cycle fluctuations in the regions as well as the asymmetries and financial imbalances within the eurozone (see for instance, Imbs, 2004; Corsetti et al., 2008; Atalay, 2017).

Kalemli-Ozcan et al. (2003) provide evidence that risk sharing and industrial specialisation are positively correlated using data that combines international and intra-regional information. The message is that countries (or regions) that can shield consumption against production risk are better equipped to exploit the gains from specialisation much more and advance further in economic integration. The channels for risk sharing, aside from fiscal transfers, are banking and capital markets integration.

Figure 1

**Employment specialisation across European regions, 2016**

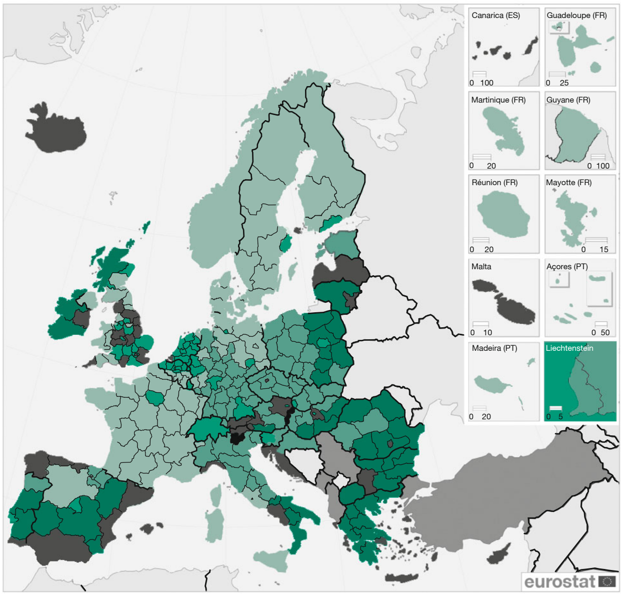

Administrative boundaries: © EuroGeographics © UN-FAO © Turkstat. Cartography: Eurostat – GISCO 5/2019.

0 200 400 600 800 km

- Agriculture, forestry and fishing
- Industry
- Construction
- Wholesale and retail trade; transport; accommodation and food services; information and communication
- Financial and insurance; real estate; professional, scientific and technical; administrative and support service activities
- Public administration (defence; social security; education; health and social work); arts, entertainment and recreation; others
- Data not available

Note: The share of the total number of persons employed in each NUTS 2 region is computed for the six activities: a similar calculation is made for the whole of the EU28; the most specialised activity is computed by taking the regional shares and subtracting the EU28 shares; the map shows for each region the activity whose employment share exceeded the EU28 average by the largest margin (as measured in percentage point terms). Norway and Switzerland: national data. Germany, Greece, Spain, France, Cyprus, the Netherlands, Poland and Romania: provisional. Slovakia: estimates.

Source: Eurostat (online data codes: nama_10r_3empers and nama_10a10_3).

Martinez et al. (2019) find that a banking union is efficient in sharing domestic demand shocks, while a capital market union is key to sharing supply shocks. That is, integration should go hand in hand on both fronts.

## An incomplete risk-sharing architecture within the EMU

The question that arises is how countries in the EU, especially those in the Economic and Monetary Union (EMU), share the risks that arise from the industrial specialisation brought by the Single Market. Hoffman et al. (2019a) find for the EMU area that, after the adoption of the euro, banking integration grew significantly through wholesale funding and the interbank market, which are highly procyclical (see Admati and Hellwig, 2014), instead of cross-border banking integration. This implies that risk sharing actually plummets during economic downturns, as we have seen during the European debt crisis in 2010-2012. As a matter of fact, sharing the same currency without advancing further in the capital market and cross-border banking integration leaves countries more exposed to asymmetric shocks and financially fragile (Jaccard and Smets, 2020), especially if those countries do not share the same banking supervision. There have been advances in banking integration (see Bénassy-Quéré et al., 2018, for a discussion on this matter) and common banking supervision, but capital market integration is still lagging. The main obstacle to the latter is that the EMU lacks a safe asset. For capital markets to become deeper, abundant safe assets are needed to provide collateral; however, as we saw during the European debt crisis, sovereign bonds of countries facing fiscal tensions lose their safe asset status and, therefore, their value as collateral (Reis, 2019).

This problem is further amplified by the so-called deadly embrace between sovereign debt and bank debt (Farhi and Tirole, 2018). The main casualty of the deadly embrace is credit to small firms, which cannot grow, hampering competition, as shown by Hoffman et al. (2019b). Thus, the implication is that, in the absence of a common safe asset, it is very difficult to break the deadly embrace and advance in cross-border banking and capital market integration, which are crucial to risk sharing.

This incomplete risk-sharing architecture is also being threatened by national responses to the pandemic. The same logic that applies to coordinating across regions within countries follows across countries in the EU, especially if we want to preserve the Schengen space. In the presence of externalities, uncoordinated actions lead to inefficient outcomes. The fact that some countries have more fiscal capacity than others implies that some countries can give more state aid to firms and sectors than other countries. This industrial policy at the member state level distorts competition and harms the Single Market, as Motta and Peitz (2020) argue. One could object that the asymmetric fiscal capacity is the result of differences in fiscal discipline, which is true. Nevertheless, we should bear in mind that tax revenues have a different elasticity across countries and, most likely, industry specialisation and the firm size distribution affect that elasticity. The estimates of Koester and Priesmeier (2017), and Mourre and Princen (2019) go in that direction.

The need for a safe common asset leads us, inevitably, to discussions of some sort of eurobonds. Mutualisation of debt among sovereign countries is not a good mechanism because it is plagued by many frictions, particularly limited commitment. But inaction is not an option because the current faulty architecture creates many economic and political tensions that endanger the European project. Additionally, faulty design can have unintended consequences. For instance, during the European debt crisis and the ensuing flight to safety, the return to German bonds reached historically negative levels. The question that arises is whether this was due to the fact that it was perceived as the only safe asset in euros. Finally, if we all agree that in order to make progress in banking and capital market integration, we need a safe asset, then we need to discuss common fiscal capacity as also argued by Pisani-Ferry et al. (2013). A safe asset will be valued if it is backed by tax revenues.

### A common fiscal instrument conciliates risk sharing and fiscal discipline

The current coronavirus crisis has shown that uncoordinated policy responses lead not only to inefficient outcomes, but also to political tensions that give rise to anti-euro populism that may threaten the European project. A very stark example is the lack of coordination in health programmes and the protectionist reactions at the beginning of the pandemic. The disruption of global supply chains provoked by the virus has changed our understanding of the meaning of ‘strategic industries’ and there are calls to reduce country specialisations in a particular sector as a way to shield the economy against some shocks; that is, to reduce regional exposure to risk instead of sharing it. The underlying logic of this argument is that EU risk sharing mechanisms do not work well when needed so that it is better to smooth income by diversifying sectoral activities within countries. Thus, there is a serious protectionist threat that we have to deactivate.

The European Union is facing two big challenges: COVID-19 and climate change. They are both negative externalities; that is, both challenges call for coordinated action. We need to deepen risk sharing by means of common fiscal capacity and, at the same time, to coordinate policy against COVID-19 and the recovery phase of our economies while acting against climate change. But in doing so, we do not want to harm incentives: we do not want an enhanced EU fiscal capacity to backfire and result in the reduced fiscal discipline of EU member states. Thus, the simplest solution is to create a common fiscal instrument to finance a common policy that should be discussed and designed in the common institutions. The EU already has the instruments to channel targeted policies across regions: the European Social Fund, the European Regional Development Fund, Horizon 2020, etc., and the European

Semester as the coordination net. This measure would complement the Stability and Growth Pact and the European Stability Mechanism.

The first candidate for the common instrument is taxes directed at firms. There are three reasons, at least, why firm taxation should be done at the European level. First, the fact that firms can move headquarters and production plants easily across countries creates a problem for fiscal competition. Second, coordinated industrial policy can be undone by taxation at the national level. And third, the very size of many companies may give them strong influence at the national level but much less influence at the EU level.

### EU common taxes to boost the Single Market

The first step would be creating a common digital service tax. The project "Fair Taxation of the Digital Economy" (European Commission, 2018) includes two proposals: a reform of corporate tax rules so that firm profits are taxed where they accrue and an interim tax that covers digital activities currently untaxed in the EU. The important thing about this initiative is that it is targeting income that is not currently taxed. That is, it is aiming to solve a problem of horizontal justice as well as efficiency, since not taxing activities distorts competition. As discussed above, the COVID-19 crisis has shifted household expenditures from face-to-face services to digital services. This huge shock has been positive for digital firms. The European Commission estimates that a 3% tax on digital services would generate €5 billion. To put this amount in perspective, notice that if the Commission raises €500 billion issuing bonds with a real return of 1%, the annual service would be exactly those €5 billion.

The second step would be to advance the Common Consolidated Corporate Tax Base (European Commission, 2016). Cross-border firms (and our goal is that EU firms grow enough to operate across borders) face 28 tax codes and have many opportunities for shifting profits and using loopholes. This fragmentation is a barrier to the Single Market, especially for small and medium-sized firms that want to grow as it is also an inefficient way to tax firms. According to the Commission (2016), 70% of firms' profit is shifted across EU member states solely for tax purposes. This tax competition leads to a race to the bottom that is inefficient for two reasons. First, because it puts the burden of taxation on human capital accumulation. Second, without tax revenues we cannot build common public goods which are essential to fight the pandemic and climate change. According to the Commission estimates, EU businesses could cut their compliance costs by 2.5% under the common base and even more with the full consolidation of the tax base. Corporate taxes collected 2.7% of GDP across the EU in 2017 (see European Commission 2019). Collecting this revenue could be done in a much more efficient way. A bolder step would be to phase out direct country contributions to the EU budget that are subject to much bargaining and negotiation and replace them gradually with revenues from the common corporate tax.

Additionally, to boost a green recovery, the EU Emissions Trading System needs to be clarified and, perhaps, allowances should be restricted. The auction system should be revised taking into account the dynamic responses of firms. We should also consider introducing a carbon tax, which levied upstream on the fuel itself when it is extracted or imported, simplifies its administration and the management of carbon leakages. These two ways of taxing firms should be coordinated within the common corporate tax base.

### The political economy of risk sharing

Finally, we should take into account the fact that the gains of improving risk sharing across EU members and the costs of reforming current rules may occur at different horizons. Economies are dynamic and change over time. Not only that, the fact that countries have heterogeneous sectors and different income and wealth distributions implies that there are always winners and losers within countries. Thus, we should study further the gains of improving the mechanisms of risk sharing across countries, taking into account the underlying heterogeneity within EU country member states. In particular, the unanimity rule in the Council of the EU is a distortion to risk sharing since many side payments, transfers or budget rebates are agreed to circumvent veto power of member states regardless of efficiency criteria. We should replace the unanimity rule with a qualified majority rule. In this way we can design better policies so the net gains of risk sharing are felt by all citizens within the European Union.
